# Benign Leydig cell tumor presenting in a solitary testis with cryptorchidism: A case report

**DOI:** 10.1016/j.eucr.2025.103232

**Published:** 2025-10-01

**Authors:** Farzad Allameh, Sina Samenezhad, Lena Yaghoubpour, Amirhossein ghasemzade

**Affiliations:** a Men's Health & Reproductive Health Research Center, Shahid Beheshti University of Medical Sciences, Tehran, Iran; b Urology Department, Shohada-e-Tajrish Hospital, Shahid Beheshti University of Medical Sciences, Tehran, Iran; c Department of Pathology, Shohada-e-tajrish Educational Hospital, School of Medicine, Shahid Beheshti University of Medical Sciences, Tehran, Iran

**Keywords:** Leydig cell tumor, Testis-sparing surgery, Sex cord-Gonadal stromal tumors, Histopathology

## Abstract

Leydig cell tumors (LCTs) are uncommon testicular neoplasms, accounting for 1–3 % of cases. While most are benign, a small subset demonstrates malignant potential, making accurate diagnosis and tailored management essential. We report the case of a 52-year-old man with a history of cryptorchidism and prior orchiectomy who presented with inguinal pain and infertility. Imaging revealed a solitary testis with a small intratesticular lesion. Intraoperative frozen section suggested a Leydig cell tumor, and partial orchiectomy was performed. Final pathology confirmed a benign LCT. The patient remains recurrence-free at follow-up, highlighting the role of testis-sparing surgery in selected cases.

## Highlights

• Rare case of benign Leydig cell tumor in a solitary, cryptorchid testis.
• Left partial orchiectomy performed with hernia repair and testis repositioning.
• Histopathology and immunohistochemistry confirmed benign Leydig cell tumor.
• Testis-sparing surgery ensured hormonal preservation and recurrence-free follow-up.

## 1 Introduction

Testicular tumors account for approximately 1 %–1.5 % of all neoplasms in men and are broadly divided into germ cell tumors and sex cord–stromal tumors. Among the latter group, Leydig cell tumors (LCTs) are the most common subtype, representing only 1 %–3 % of all testicular tumors in adults.^1^^,^^2^ While rare overall, they hold clinical importance because of their diverse presentations, ranging from asymptomatic testicular masses to endocrinological manifestations such as gynecomastia, loss of libido, or infertility.^1^^,^^2^

Although most LCTs follow a benign course, up to 10 % may behave malignantly, with metastatic potential to the lymph nodes, lungs, liver, and bones.^1^, ^2^, ^3^ Clinical and pathological predictors of malignancy include older age at presentation, tumor size >5 cm, infiltrative margins, vascular invasion, necrosis, and increased mitotic activity.^1^^,^^2^ Nevertheless, the definitive diagnosis relies on histopathology and immunohistochemistry, as imaging features often overlap with those of germ cell tumors.^1^^,^^2^^,^^4^ Typically, LCTs demonstrate positivity for inhibin and calretinin, while tumor markers such as α-fetoprotein (AFP) and β-human chorionic gonadotropin (β-hCG) remain negative, aiding their distinction from germ cell malignancies.^2^^,^^4^

In recent years, incidental detection of LCTs has increased due to widespread use of scrotal ultrasound in the evaluation of infertility and other testicular conditions.

^2^^,^^5^. Indeed, associations with cryptorchidism, infertility, and gynecomastia suggest that LCTs may arise within the spectrum of testicular dysgenesis syndrome.^5^ The strong link between LCTs and endocrine dysfunction underscores the need for careful hormonal evaluation, both at diagnosis and during follow-up.^2^^,^^5^

Management of LCTs depends on tumor characteristics and clinical context. Radical inguinal orchiectomy remains the standard of care for most cases.^1^^,^^2^^,^^4^ However, with growing recognition of their generally indolent behavior, testis-sparing surgery or even active surveillance may be considered in select, compliant patients particularly those with a solitary testis, bilateral tumors, or concerns regarding fertility preservation.^5^^,^^6^ Despite their usually favorable prognosis, the small subset of malignant LCTs respond poorly to chemotherapy or radiotherapy, making close surveillance essential.^2^^,^^3^

Here, we present the case of a middle-aged man with a history of cryptorchidism and infertility, who was ultimately diagnosed with a benign Leydig cell tumor following testis-sparing surgery. This case highlights both the diagnostic challenges and the nuanced management decisions involved in the care of patients with rare testicular stromal tumors.

## 2 Case report

A 52-year-old male with a history of left undescended testis (UDT) and a prior right inguinal orchiectomy at the age of 12, of unknown indication and pathology, presented with intermittent left-sided testicular pain described as sudden, thunder-like episodes. He had undergone left inguinal hernia repair at the age of 16. During infertility evaluation following marriage, semen analysis revealed non-obstructive azoospermia, confirmed by microdissection testicular sperm extraction (micro-TESE) of the left testis, which yielded no spermatozoa. The patient subsequently required donor sperm for in vitro fertilization. He reported preserved libido and erectile function, with no constitutional symptoms.

On examination, the left testis was not palpable in the scrotum. A firm structure was noted at the level of the left inguinal ring. Scrotal and inguinal ultrasonography demonstrated a hypoechoic structure in the left hemiscrotum, measuring 7 × 3 mm, consistent with an atrophic testis, and a 15 cc cystic lesion in the left inguinal region, suggestive of a persistent processus vaginalis. Serum tumor markers were within normal limits (LDH 37.1 U/L, β-hCG <0.1 mIU/mL, AFP 3.5 ng/mL).

Magnetic resonance imaging (MRI) revealed a left testis measuring 38× 24 mm, located at the internal inguinal ring. A well-defined 10 mm lesion was identified at the infero-anterior pole, showing T2 hypointensity, T1 isointensity with mild hyperintensity, restricted diffusion, and homogeneous hypoenhancement. There was no evidence of tunica albuginea invasion or regional lymphadenopathy. The radiologic impression favored a sex cord–stromal tumor, with seminoma considered as a differential diagnosis.

Given the solitary testis and imaging findings, surgical exploration with intraoperative frozen section was performed. Through a left inguinal incision, a suspicious testicular mass was identified adjacent to the epididymis (Fig. 1-A). En bloc resection was performed and submitted for frozen section (Fig. 1-B), which was reported as a sex cord–stromal tumor, most consistent with Leydig cell tumor. The specimen margins were grossly free of tumor at frozen section. Considering the patient's solitary testis, a partial orchiectomy was undertaken. The tunica albuginea was repaired, and the testis was lowered to the lowest possible position within the scrotum, left-sided inguinal hernia was present and repaired during surgery.

**Figure 1-A:**
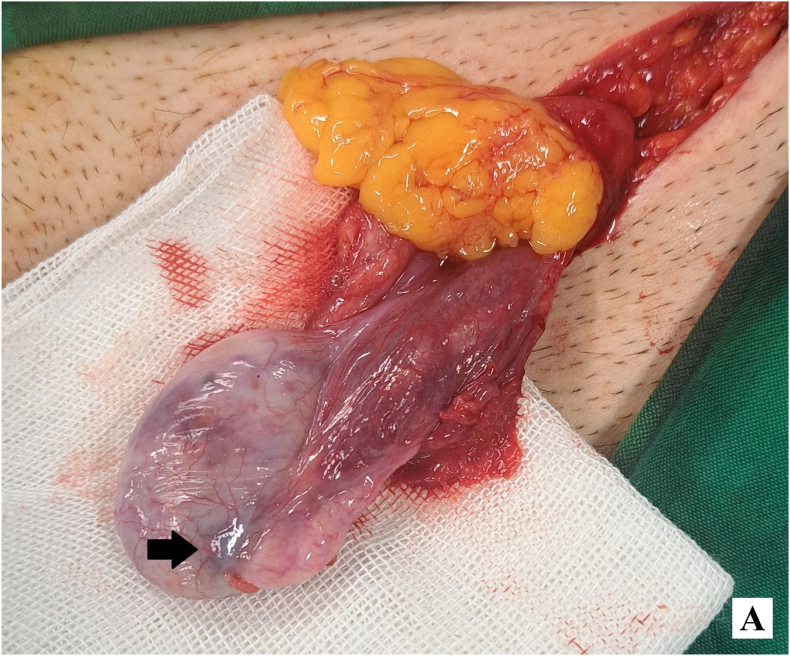
suspicious testicular mass was identified adjacent to the epididymis.

**Figure 1-B:**
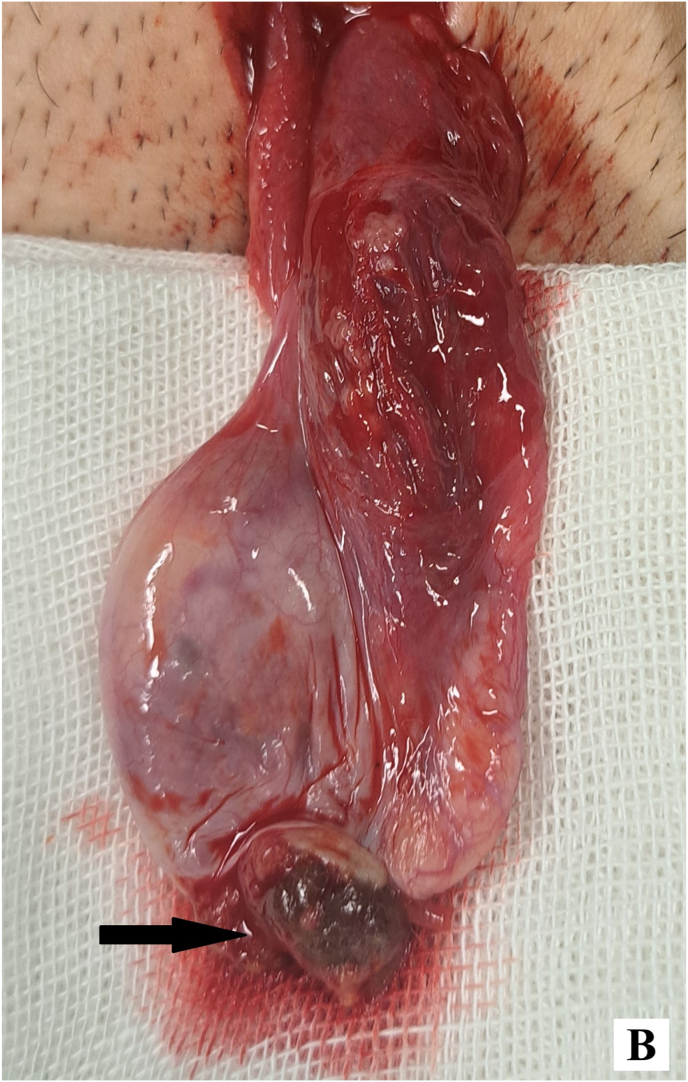
En bloc resection of Mass.

The postoperative course was uneventful, and the patient was discharged on the first postoperative day. Permanent histopathology demonstrated a neoplastic proliferation of polygonal cells with abundant eosinophilic cytoplasm, uniform round nuclei, and prominent nucleoli arranged in a diffuse pattern. Reinke crystals were not identified. Features of malignancy, including tumor size >5 cm, infiltrative borders, necrosis, vascular invasion, and high mitotic activity, were absent (Figure 2-A, Figure 2-B). Immunohistochemistry revealed cytoplasmic positivity for inhibin (Figure 3-A, Figure 3-B) and calretinin (Figure 4-A, Figure 4-B), with negative staining for WT1, confirming the diagnosis of Leydig cell tumor, pathologic stage pT1aNxMx. The separately resected scrotal tissue was unremarkable, and the epididymis was free of tumor.

**Figure 2-A:**
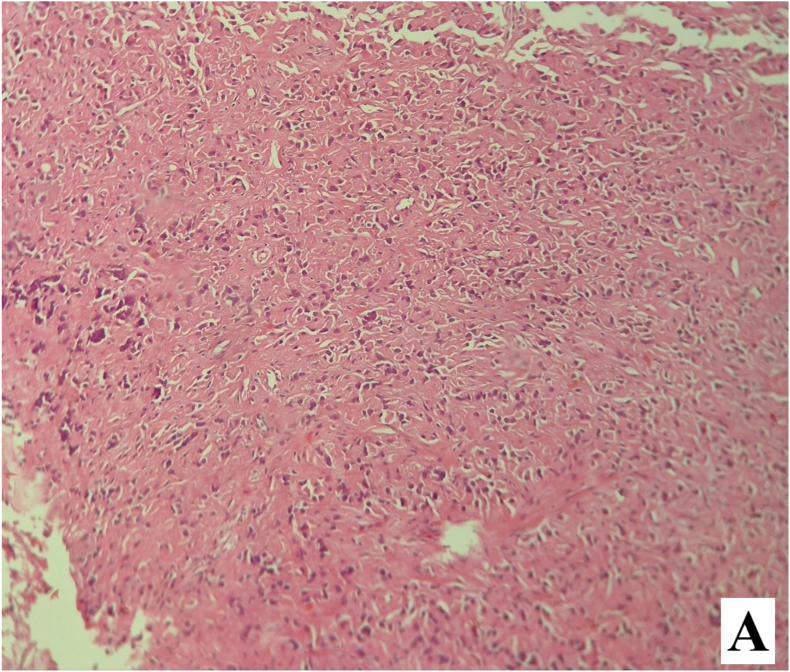
neoplastic proliferation of polygonal cells with abundant eosinophilic granular cytoplasm (10x).

**Figure 2-B:**
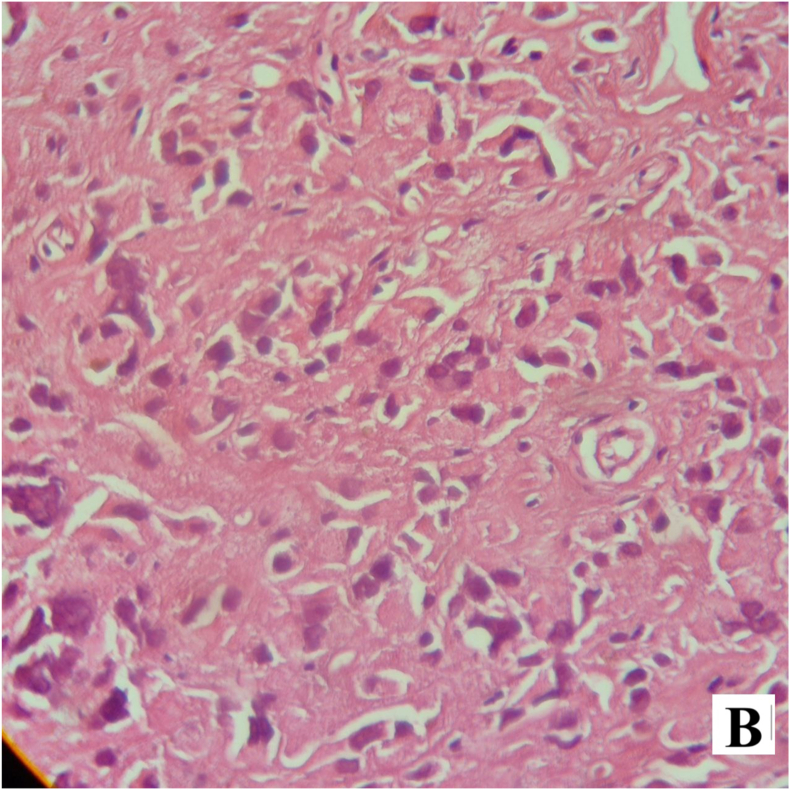
uniform round nuclei and prominent central nucleoli in diffuse architecture (40x).

**Figure 3-A:**
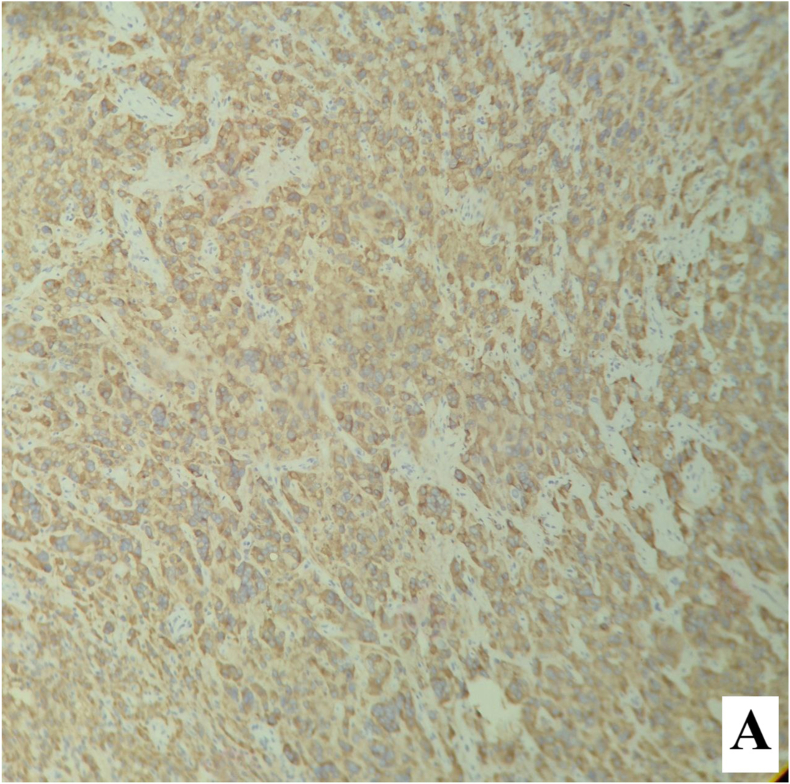
immunohistochemistry staining with inhibin.

**Figure 3-B:**
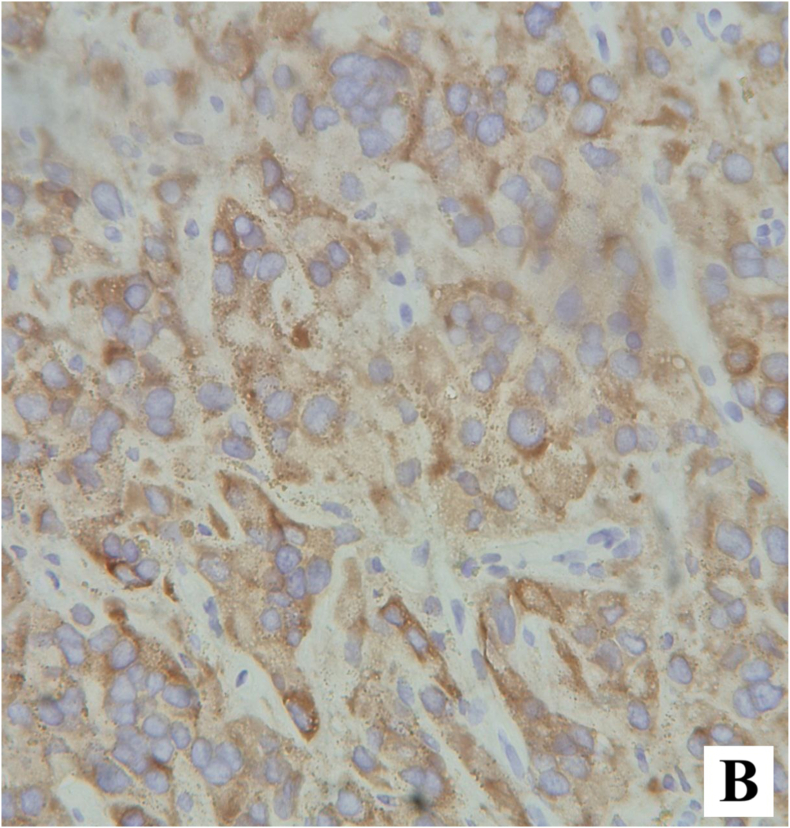
inhibin immunohistochemistry staining shows cytoplasmic positivity in tumoral cells.

**Figure 4-A:**
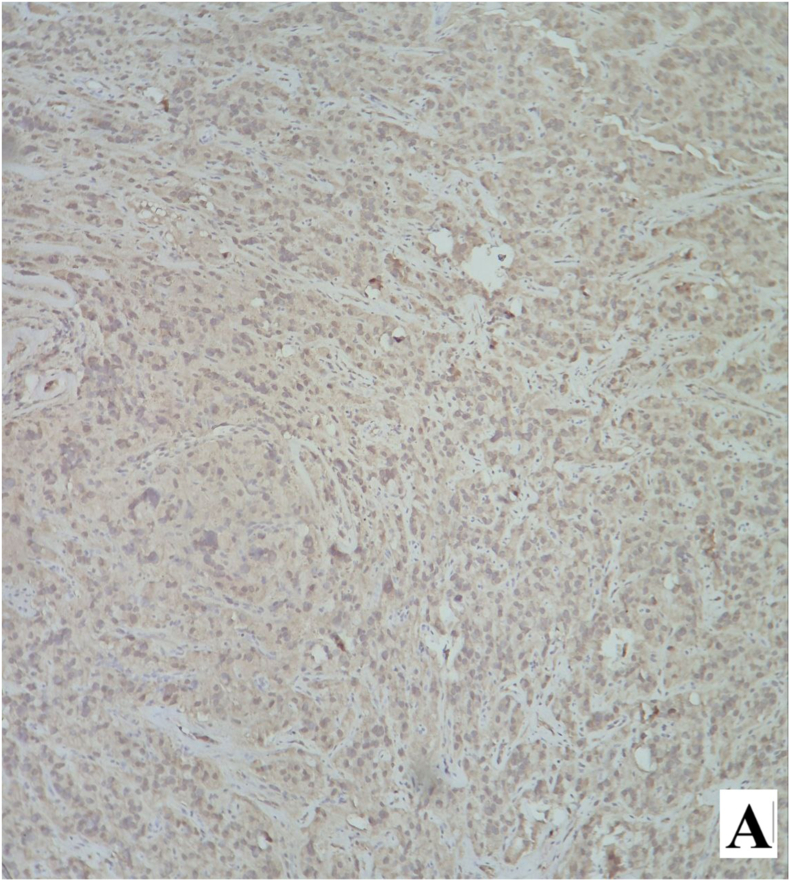
immunohistochemistry staining with calretinin shows cytoplasmic positivity in tumoral cells.

**Figure 4-B:**
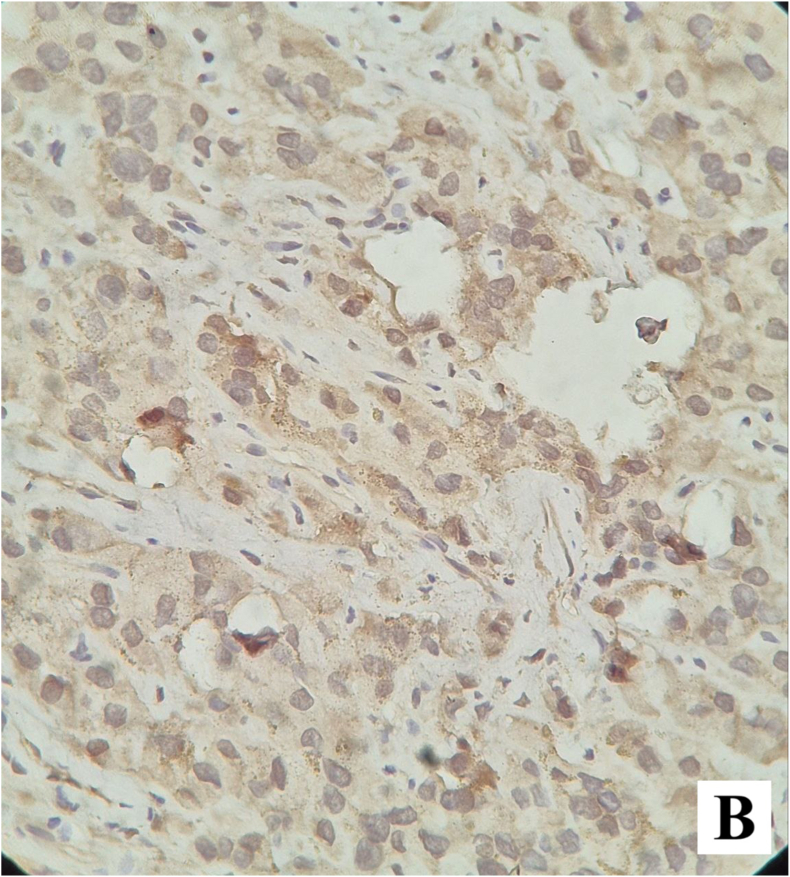
calretinin immunohistochemistry staining shows cytoplasmic positivity in tumoral cells.

At three-month follow-up, clinical examination was unremarkable. At six months, contrast-enhanced CT of the abdomen and pelvis demonstrated no evidence of recurrence or metastasis. Hormonal and laboratory evaluations remained stable. The patient remains under surveillance with planned six-monthly follow-up.

## 3 Discussion

Leydig cell tumors (LCTs) represent a small proportion of testicular neoplasms, accounting for only 1–3 % of cases.^5^^,^^7^ Although considered rare, their incidence has been increasing, partly due to the wider use of scrotal imaging.^5^^,^^8^ They are typically benign tumors, with malignancy estimated in around 2–10 % of cases,^5^, ^6^, ^7^, ^8^, ^9^ most often presenting in men between the third and sixth decades of life.^7^ Clinical features may vary; some patients remain asymptomatic, while others present with testicular pain, infertility, gynecomastia, or hormonal disturbances.^2^^,^^5^^,^^8^^,^^9^ Risk factors such as cryptorchidism, gynecomastia, and testicular dysgenesis are frequently associated, supporting a link between LCTs and impaired testicular development.^5^^,^^9^

Histologically, most LCTs are benign and carry an excellent prognosis.^1^^,^^7^^,^^8^ Malignant transformation is rare, but when present, it carries a significant risk of metastasis, which underscores the importance of careful histopathological evaluation and long-term surveillance.^1^^,^^8^^,^^9^ Even in benign cases, endocrine abnormalities and impaired spermatogenesis may persist after tumor removal, highlighting the need for ongoing follow-up and fertility counseling.^2^^,^^5^^,^^9^

Traditionally, radical orchiectomy was considered the standard treatment, regardless of tumor size or laterality.^4^ However, emerging evidence has shown that testis-sparing surgery can be a safe and effective alternative in well-selected patients, particularly when the contralateral testis is absent or compromised.^5^^,^^7^^,^^10^^,^^11^ Organ-preserving approaches offer the advantage of maintaining endocrine function and fertility potential without compromising oncological safety, provided that close surveillance protocols are followed.^10^^,^^11^

In our patient, the decision to perform a partial orchiectomy was strongly justified by the presence of a solitary testis. This strategy successfully avoided the need for lifelong hormonal replacement therapy and helped preserve sexual function and overall quality of life. Given the benign nature of most LCTs and the low risk of recurrence after adequate excision, conservative management with testis-sparing surgery combined with regular clinical and radiological monitoring represents an appropriate and patient-centered approach. Nonetheless, the relatively short follow-up period in our case remains a limitation, and longer surveillance is necessary to fully confirm the long-term oncological safety of this approach.

## 4 Conclusion

Leydig cell tumors are rare testicular neoplasms. This case is notable for the coexistence of cryptorchidism, a solitary testis, and the successful use of a testis-sparing approach. It highlights the importance of individualized management strategies that balance oncological safety with the preservation of hormonal function and fertility potential.

## CRediT authorship contribution statement

**Farzad Allameh:** Writing – review & editing, Validation, Supervision, Methodology, Investigation, Conceptualization. **Sina Samenezhad:** Writing – original draft, Visualization, Validation, Resources, Project administration, Methodology, Investigation, Data curation, Conceptualization. **Lena Yaghoubpour:** Visualization, Resources, Data curation. **Amirhossein ghasemzade:** Visualization.

## Patient consent

Patient informed consent was obtained to publish his information. The patient's private information remained confidential with the researchers.

## Ethical approval

This study was reviewed by our hospital Institutional Review Board (IRB) and was deemed exempt from formal ethical approval because the treatment and data collection were based entirely on established clinical guidelines and standard urological practice performed by an expert urologist. No experimental interventions or novel protocols were involved, and all actions followed evidence-based, routine medical care.

## Funding

This research received no specific grant from any funding agency in the public, commercial, or not-for-profit sectors.

## Declaration of competing interest

The authors declare no competing financial or personal interests.
